# Biological Characteristics of Fluorescent Superparamagnetic Iron Oxide Labeled Human Dental Pulp Stem Cells

**DOI:** 10.1155/2017/4837503

**Published:** 2017-02-16

**Authors:** Liang Ma, Ming-wei Li, Yu Bai, Hui-hui Guo, Sheng-chao Wang, Qing Yu

**Affiliations:** ^1^State Key Laboratory of Military Stomatology & National Clinical Research Center for Oral Diseases & Shaanxi Key Laboratory of Oral Diseases, Department of Operative Dentistry and Endodontics, The Fourth Military Medical University, Xi'an, China; ^2^Department of Stomatology, No. 44 Hospital of Chinese PLA, Guiyang, Guizhou, China; ^3^State Key Laboratory of Military Stomatology & National Clinical Research Center for Oral Diseases & Shaanxi Key Laboratory of Oral Diseases, Department of Preventive Dentistry, School of Stomatology, The Fourth Military Medical University, Xi'an, China

## Abstract

Tracking transplanted stem cells is necessary to clarify cellular properties and improve transplantation success. In this study, we investigate the effects of fluorescent superparamagnetic iron oxide particles (SPIO) (Molday ION Rhodamine-B™, MIRB) on biological properties of human dental pulp stem cells (hDPSCs) and monitor hDPSCs in vitro and in vivo using magnetic resonance imaging (MRI). Morphological analysis showed that intracellular MIRB particles were distributed in the cytoplasm surrounding the nuclei of hDPSCs. 12.5–100 *μ*g/mL MIRB all resulted in 100% labeling efficiency. MTT showed that 12.5–50 *μ*g/mL MIRB could promote cell proliferation and MIRB over 100 *μ*g/mL exhibited toxic effect on hDPSCs. In vitro MRI showed that 1 × 10^6^ cells labeled with various concentrations of MIRB (12.5–100 *μ*g/mL) could be visualized. In vivo MRI showed that transplanted cells could be clearly visualized up to 60 days after transplantation. These results suggest that 12.5–50 *μ*g/mL MIRB is a safe range for labeling hDPSCs. MIRB labeled hDPSCs cell can be visualized by MRI in vitro and in vivo. These data demonstrate that MIRB is a promising candidate for hDPSCs tracking in hDPSCs based dental pulp regeneration therapy.

## 1. Introduction

Human dental pulp stem cells (hDPSCs), firstly isolated from adult human third molar dental pulp, have been described to be possess self-renewal capacity, high proliferation potential, and the ability to undergo multilineage differentiation [1]. Compared with other adult stem cells, hDPSCs exhibit a stronger proliferation potential and dentinogenic ability [2]. Recent study reported strong neurogenic potential as hDPSCs highly expressed III *β*-tubulin (TUBB3) and microtubule-associated protein 2 (MAP2) under inductive condition [3]. Furthermore, it has been shown that hDPSCs seem to be a particularly good choice for reconstruction of different craniomaxillofacial tissues and organs, such as cranial bones, nerves, teeth, and salivary glands [4]. Taken together, these studies provide sufficient evidence to regard hDPSCs as an important candidate for future cell-based clinical applications.

For a successful stem cell therapy, an underlying prerequisite is survival and appropriate localization of the transplanted stem cells [5]. Therefore, cell tracking in vivo is important for the development of successful stem cell therapies. With superior resolution and localization, magnetic resonance imaging (MRI) has emerged as the leading modality for tracking transplanted stem cells in living animals and clinical studies [6]. The advantages of MRI are that it is noninvasive and is suitable for longitudinal studies [7]. To distinguish specific cells using MRI, transplanted cells must be labeled with a magnetic contrast agent. In recent years, various magnetic nanoparticles, such as superparamagnetic iron oxide (SPIO), have been widely applied both in cell tracking and in magnetic targeting [8–10]. However, before being applied for both experimental animals and eventually clinical use, the biological impacts of different kinds of SPIO on different types of stem cells must be investigated.

Currently, MIRB (Molday ION Rhodamine B; BioPAL, Worcester, MA, USA), as a new USPIO agent, has become a new research hot spot in stem cell labeling and tracking [11–13]. Compared with other SPIOs, it has higher labeling efficiency and can be internalized by stem cells without the use of adjuvant transfection agents and can be visualized by both MRI and fluorescence microscopy [14]. A variety of cell types have been successfully labeled with MIRB and their proliferation, phenotype, and differentiation after labeling were investigated [5, 6, 12, 14, 15]. However, little is known about the biological impacts of MIRB on hDPSCs. In this study, we characterized the labeling and loading properties of MIRB on hDPSCs. Meanwhile, the cell proliferation, apoptosis, and odonto-/osteogenic differentiation were qualitatively and quantitatively analyzed. Furthermore, we established a dental pulp ectopic regeneration model using hDPSCs cell sheet and immunodeficiency mice and then assessed the potential for imaging and monitoring of transplanted cell sheet by MRI in vivo.

## 2. Methods

### 2.1. Isolation and Culture of Human Dental Pulp Stem Cells (hDPSCs)

Dental pulp stem cells were isolated as previously described by Gronthos et al. [1]. Primary human dental pulp stem cells were isolated from the third molars obtained from patients undergoing extraction. All the procedures were performed under the approval of Ethics Committee of the Fourth Military Medical University. Informed consents were obtained from all subjects. Briefly, freshly extracted teeth were collected from patients ranging from 18 to 23 years old and used for cell isolation within 1 hour. The pulp tissues removed from the teeth were cut into small pieces and digested in a solution with 3 mg/mL collagenase type I and 4 mg/mL dispase (both from Sigma-Aldrich, St. Louis, MO, USA) for 30 to 60 min at 37°C. Afterward, the digested mixtures were seeded in six-well plates (Corning Costar, Rochester, NY, USA), cultured with *α*-MEM supplemented with 20% fetal bovine serum (FBS; Hyclone), 100 U/mL penicillin-G and 100 mg/mL streptomycin (both from Roche, Basel, Switzerland), and maintained in an atmosphere of 5% CO_2_ at 37°C. The medium was changed every 2 days. When reaching 60% confluence, the cells were trypsinized to obtain single cell clones (passage 0) by limiting dilution. Cells from passage 3 to 5 were used for this study.

### 2.2. MIRB hDPSCs Loading Characterization

#### 2.2.1. Prussian Blue Staining

For confirmation of cellular iron uptake, passage 3 (P3) hDPSCs were plated in 24-well plates at an initial density of 3 × 10^4^ cells/well and incubated at 37°C with 5% CO_2_. Molday ION Rhodamine B (MIRB, BioPAL Co., Worcester, MA, USA) stock solution (2 mg Fe/mL) was added to the cell-culture medium (*α*-MEM + 10% FBS) at the final concentrations: 0, 12.5, 25, 50, and 100 *μ*g Fe/mL and hDPSCs were incubated for 24 h under standard culture conditions (37°C, 5% humidified CO_2_). After incubation, the MIRB-containing medium was removed by aspiration and hDPSCs were washed twice with phosphate buffered saline (PBS, Hyclone) to remove extracellular MIRB. Then the cells were fixed with 4% paraformaldehyde (PFA) for 30 min and then stained with Perl's reagent (2% potassium ferrocyanide in 6% hydrochloric acid) for 30 min to evaluate intracellular Fe distribution by light microscopy.

#### 2.2.2. Fluorescence Microscopy

Intracellular distribution of red fluorescent MIRB nanoparticles after labeling was observed by fluorescence microscopy as previously described by Xiao et al. [16]. Briefly, cells were seeded on glass bottom cell culture dish (Φ 15 mm, NEST, Wuxi, China) at an initial density of 3 × 10^4^ cells/dish. After being labeled at various concentrations of MIRB mentioned above for 24 h, cells were washed with ice-cold PBS and fixed with ice-cold 4% paraformaldehyde. After being washed with PBS for three times, the cells were permeabilized in 0.1% Triton X-100 for 15 min and blocked for 1 h with 3% BSA in PBS at room temperature. Filamentous actin was stained with 320 nmol/L FITC-phalloidin conjugate solution (Sigma) in PBS for 2 h at 4°C. After several washes in PBS, cells were observed under laser confocal microscopy (Olympus, Japan).

#### 2.2.3. Electron Microscopy

The distribution of the MIRB particles within the cells was examined under transmission electron microscopy (TEM: JEM1230; Jeol Ltd., Tokyo, Japan). Harvested labeled and unlabeled hDPSCs were immersed in 2.5% buffered glutaraldehyde at 4°C for 1 h and fixed with 1% osmium tetroxide (Fluka, Sigma-Aldrich) for 2 h for observation.

#### 2.2.4. Labeling Efficiency

To quantitatively evaluate the labeling efficiency, hDPSCs were assessed by counting cells positive for Prussian blue staining and the presence of Rh-B, which indicated MIRB presence within the cell. Briefly, 10 fields of view were randomly chosen for counting Prussian blue positive cells under 10 times microscope, and the labeling efficiency was calculated by following equation: labeling efficiency = (Prussian blue positive cell number/whole cell number) × 100%.

#### 2.2.5. Fe Uptake Quantification

Average cellular MIRB uptake was determined using an iron assay kit (Biovision, Inc., CA, USA) according to the manufacturer's instructions. Briefly, labeled hDPSCs from 6-well plates were lysed in 65 *μ*L Iron Assay Buffer, centrifuged at 16 000*g* for 10 minutes to remove insoluble materials. Fifty microliters of the supernatant from each sample was added to sample wells in a 96-well plate and the volume was brought to 100 *μ*l/well with Assay Buffer. Then, 5 *μ*L iron reducer was added to each sample to reduce Iron (III) to Iron (II). Standard curve was established according to instruction; then iron standards and samples were incubated for 30 min at 25°C. After that, 100 *μ*L Iron Probe was added to each well and iron standard and samples were incubated for 60 min at 25°C, protected from light. Absorbance was read with a spectrometer at 593 nm. Total Fe per hDPSCs was calculated by dividing total Fe per well by counted hDPSCs per well.

### 2.3. Cell Proliferation

Cell proliferation was evaluated by MTT assay. Cells were seeded in 96-well plate at a density of 1 × 10^3^ cells per well with 100 *μ*L medium (37°C and 5% CO_2_) and allowed to attach overnight. Then the medium was removed and replaced with fresh medium containing varying concentrations of MIRB (12.5 *μ*g/mL, 25 *μ*g/mL, 50 *μ*g/mL, and 100 *μ*g/mL, resp.) for 1 d, 3 d, 5 d, and 7 d. Wells containing only the culture medium served as control. The cells were incubated in 100 *μ*L of medium with 20 *μ*L of 5 mg/mL MTT solution for 4 h at 37°C. After removing the medium containing MTT, 150 *μ*L dimethyl sulfoxide (DMSO) was added to each well to dissolve the formazan. The absorbance was read at 490 nm using a microplate reader (Model 680; Bio-Rad-Benchmark, Hercules, CA, USA).

### 2.4. Cell Epitope Pattern Analysis

MIRB-labeled (12.5 *μ*g Fe/mL) and unlabeled hDPSCs were collected and washed with cold PBS twice and then resuspended in PBS containing 1% bovine serum albumin (BSA) at 4°C and stained with fluorescent antibodies at 4°C for 30 min. The presence of MSCs surface markers, CD29, CD44, CD90, and CD105, as well as the absence of cell surface markers typical of lymphocytic and hematopoietic cells, CD34 and CD45, was analyzed using a FACS Calibur flow cytometer (Beckman Coulter Epics XL.MCL, Fullerton city, CA, USA).

### 2.5. Cell Cycle Distribution and Apoptosis Assessment

Cell cycle distribution analysis was performed as previously described [17]. HDPSCs were seeded at 5 × 10^5^ per well in 6-well plate and labeled by different concentrations of MIRB (12.5 *μ*g/mL, 25 *μ*g/mL, 50 *μ*g/mL, and 100 *μ*g/mL) for 24 h. MIRB-labeled and unlabeled hDPSCs (1 × 10^6^ cells for each) were harvested by trypsinization and fixed with 2 mL of 70% ice-cold ethanol overnight at 4°C. After centrifugation, the cells were resuspended in 2 mL of PBS and filtered using a 200-mesh cell screen. The pelleted cells were resuspended in 1 mL of 50 *μ*g/mL PI solution containing 20 *μ*g/mL RNase. The cells were incubated in the dark for 1 h at 4°C, scanned using a FACSCalibur flow cytometer (Beckman Coulter Epics XL.MCL, Fullerton city, CA, USA), and analyzed using the Multicycle software. Apoptosis effect of MIRB labeling on hDPSCs was performed using Annexin V-FITC Apoptosis Detection Kit (Keygen Biotech, Nanjing, China). The cells were labeled as mentioned above and harvested. Subsequently, 1 × 10^6^ cells from each group were stained with 5 *μ*L of Annexin V-FITC and 5 *μ*L of PI in a 500 *μ*L binding buffer for 15 min at room temperature in the dark. The apoptotic cells were then determined using a FACSCalibur flow cytometer (Beckman Coulter Epics XL.MCL, Fullerton city, CA, USA) and analyzed using the EXPO32 software.

### 2.6. Odonto-/Osteogenic Differentiation

Odonto-/osteogenic differentiation was measured to assess the effect of MIRB on the transdifferentiation potential of hDPSCs as previously described by Wang et al. [8] using differentiation medium (Cyagen Biosciences Inc., Santa Clara, CA, USA). For osteogenic differentiation, hDPSCs were seeded at 3.1 × 10^3^ cells/cm^2^ and labeled by different concentrations of MIRB (12.5 *μ*g/mL, 25 *μ*g/mL, and 50 *μ*g/mL) and then allowed to reach 90% confluence. Then MIRB-labeled and unlabeled hDPSCs were cultured in OsteoDiff Medium and the medium was changed every three days. Seven days and 14 days later, the alkaline phosphatase staining kit (Jiancheng Bioengineering Institute, Nanjing, China) was used to detect alkaline phosphatase (ALP), and 21 days later, the 1% alizarin red staining (Sigma, St. Louis, MO, USA) was performed to detect the Ca nodules.

### 2.7. Real-Time-Polymerase Chain Reaction (RT-PCR)

After osteogenic differentiation for 7 days and 14 days, the expressions of important osteogenesis-related genes, including alkaline phosphatase (ALP), bone sialoprotein (BSP), dentin sialophosphoprotein (DSPP), and osteocalcin (OCN), were detected by RT-PCR as previously described [18]. The RNA of hDPSCs with and without MIRB labeling (12.5 *μ*g/mL) was isolated using RNAiso Plus (Takara, Japan). Reverse transcription was performed using a PrimeScript RT reagent Kit (Takara). Primers were designed as follows:

ALP primers were sense, 5′-CCACGTCTTCACATTTGGTG-3′; and antisense, 5′-AGACTGCGCCTGGTAGTTGT-3′.

BSP primers were sense, 5′-AAAGTGAGAACGGGGAACCT-3′; and antisense, 5′-GATGCAAAGCCAGAATGGAT-3′.

DSPP primers were sense, 5′-GCATTTGGGCAGTAGCATGG-3′; and antisense, 5′-CTGACACATTTGATCTTGCTAGGAG-3′.

OCN primers were 5′-TGAGAGCCCTCACACTCCTC-3′; and antisense, 5′-ACCTTTGCTGGACTCTGCAC-3′.

GAPDH primers were 5′-GCACCGTCAAGGCTGAGAAC-3′; and antisense, 5′-TGGTGAAGACGCCAGTGGA-3′.

RT-PCR was performed using an ABI PRISM 7500 Real-Time PCR thermocycler (Applied Biosystems, Foster City, CA, USA). Gene expression was quantified using the SYBR Premix Ex Taq™ kit according to the manufacturer's protocol. Reactions involved were primary denaturation at 95°C for 30 s followed by 40 cycles at 95°C for 5 s and 60°C for 34 s. Relative mRNA expression was determined after normalizing the cycle threshold values for each gene with the internal control (GAPDH).

### 2.8. In Vitro Magnetic Resonance Imaging of MIRB-Labeled hDPSCs

HDPSCs labeled with different concentrations of MIRB (12.5 *μ*g/mL–100 *μ*g/mL) were harvested and counted with a hemacytometer. 1 × 10^5^ or 1 × 10^6^ cells from each group were transferred into 1.5-ml microcentrifuge tubes (Eppendorf, Westbury, NY, USA). After being centrifuged at 150 ×g for 5 min, the hDPSCs pellets at the bottom of each tube were fixed with 4% PFA for 15 min and then the PFA was discarded. Then 1% (w/v) agarose solution was added into each tube which was kept at 4°C overnight for solidification. Then the tubes were imaged with an eight-channel phased-array head coil on a 3.0-Tesla MR scanner (Siemens, Germany). Spin Echo T2-weighted (SE T2WI) images were taken (repetition time [*T*_*R*_] = 4,000 ms, echo time [*T*_*E*_] = 89 ms, flip angle = 120, field of view [FOV] = 150 × 150 mm^2^, slice thickness = 4 mm, Mat: 256 × 256).

### 2.9. In Vivo MRI of MIRB-Labeled hDPSCs in the Root Fragments of Human Teeth

#### 2.9.1. Animal Model Establishment

To monitor the transplanted hDPSCs noninvasively during pulp regeneration using cell sheet strategy in vivo, a model system that includes preparing hDPSCs cell sheets, human root segments, and immunocompromised mice was used according to the previous study with minor modification [19]. The preparation of root fragments of human teeth was performed according to the reported methods [20]. The insertion of cell sheets into root fragments was performed as follows. Briefly, hDPSCs at passage 3 were seeded on culture dishes (60 mm in diameter) at a cell density of 5 × 10^5^ cells/dish and then labeled with MIRB (12.5 *μ*g/mL). The MIRB-labeled and unlabeled hDPSCs were cultured and allowed to reach 90% confluence and the medium was changed into cell sheet induction medium (*α*-MEM + 10 *μ*g/mL vitamin C + 10% FBS). Fourteen days later, the cell sheets were harvested and were inserted into the canal space of each root fragment and kept in the wells of 12-well plates with a minimal amount of cell culture medium. Then nine 6-week-old male nude mice (body weight 18–22 g) were used. Animal experiments were approved by Experimental Animal Board of the Fourth Military Medical University. Animals were randomly divided into three groups; each group contained three mice. The animals were anaesthetized with 30 mg/mL pentobarbital sodium (30 mg/kg body wt) and then disinfected. After that, the subcutaneous implantation of two root fragments which contained MIRB-labeled and unlabeled cell sheet was performed, respectively.

#### 2.9.2. MRI Analysis

MRI of anesthetized nude mice was performed immediately after transplantation and then at 30 and 60 days after transplantation. Mice were anesthetized with 30 mg/mL pentobarbital sodium and then imaged with a customized mice coil (CG-MUC18-H300-AS, Chen Guang medical technology Co., Ltd, Shanghai, China) on a 3.0-Tesla MR scanner (Siemens, Germany). Spin Echo T2-weighted (SE T2WI) images were taken (repetition time [*T*_*R*_] = 3,500 ms, echo time [*T*_*E*_] = 89 ms, flip angle = 120, field of view [FOV] = 64 × 64 mm^2^, slice thickness = 0.8 mm, Mat: 256 × 256).

#### 2.9.3. Image Analysis and Quantification

Quantitative analysis of the signal intensity of MIRB was conducted using IPP (version 6.0, Media Cybernetics, Silver Spring, MD, USA). Initially, the color of original MRI images of all mice was reversed and the following parameters were set for the area of interest: R, 0–255; G, 0–255; B, 0–255. Then the average integrated optical density values (IOD) for the area of interest, which represented the intensity of MIRB, were measured.

#### 2.9.4. Histological Analysis

After MRI analysis, the anesthetized nude mice were sacrificed 0 d, 1 month, and 2 months, separately. The implanted root fragments were harvested and fixed by 4% paraformaldehyde (PFA) at 4°C overnight. All specimens were decalcified in 10% EDTA containing 2.5% PFA for 6–8 weeks, dehydrated, embedded in paraffin, and serially sectioned at 5 *μ*m. Then Prussian blue staining was performed to evaluate the change of iron content as a function of time.

### 2.10. Statistical Analysis

All data are expressed as mean ± standard deviation (SD). One-way analysis of variance (ANOVA) was performed to examine the effect of differing concentrations of MIRB on cell proliferation, cell cycle, apoptosis, and osteogenic differentiation. All statistical analyses were performed using the SPSS software package (version 17.0; SPSS, Inc., Chicago, IL, USA). A value of *P* < 0.05 was considered as statistically significant.

## 3. Results

### 3.1. The Isolation and Characterization of Human DPSCs

**Figure 1:**
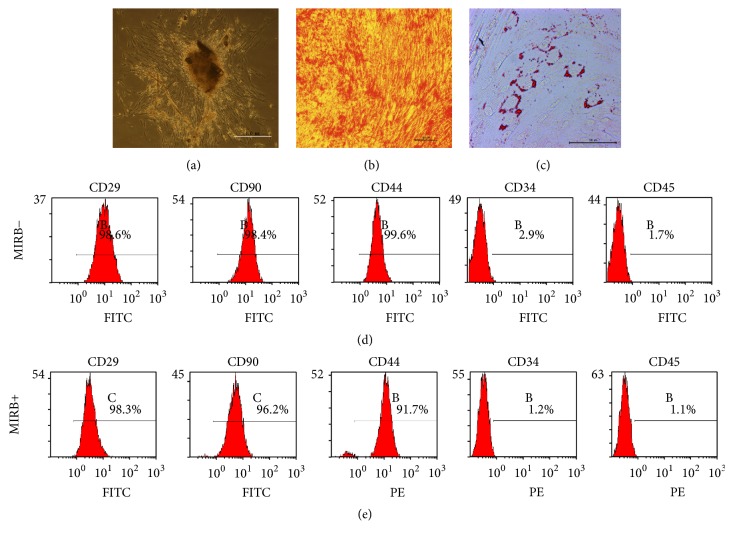
Isolation and characterization of human dental pulp stem cells (DPSCs). (a) The morphological observation of primary culture expanded dental pulp stem cells (DPSCs). (b) Odontogenic/osteogenic differentiation of DPSCs. (c) Adipogenic differentiation of DPSCs. (d and e) Representative flow cytometry analysis of cell surface markers in unlabeled and labeled hDPSCs. Cell surface markers (d) on unlabeled hDPSCs in P3 and (e) on MIRB-labeled hDPSCs in P3. Data show that both labeled and unlabeled hDPSCs are negative for CD34 and CD45 while they are positive for CD29, CD90, and CD44.

In this study, human DPSCs were successfully isolated from the pulp tissue of 6 extracted third molars. The primary cells presented clone-like growth after they were incubated for 72 h (Figure 1(a)). The flow cytometry was then performed to test the surface markers of 3rd-generation cells, namely, CD29 (98.6%), CD90 (98.4%), CD44 (99.6%), CD34 (2.9%), and CD45 (1.7%) (Figure 1(d)). In addition, the multiple lineage differentiation tests revealed that after 4 weeks of odonto-/osteogenic induction, the cells stained positive for mineral nodules with Alizarin red S (Figure 1(b)). Five weeks of adipogenic induction, the obtained cells stained positive for lipid droplets with Oil-Red O (Figure 1(c)).

### 3.2. Cell Surface Markers

To characterize the phenotype of cultured hDPSCs after MIRB-labeling, we examined the surface markers CD29, CD90, and CD44, which were present on hDPSCs, as well as an absence of CD34 and CD45 as determined by flow cytometry. The results showed that, after MIRB labeling, no significant difference existed between the phenotypic profile of MIRB-labeled and control hDPSCs at a labeling concentration of 12.5 *μ*g/mL MIRB (Figure 1(e)).

### 3.3. Morphological Observation of MIRB-Labeled Cells and Labeling Efficiency

**Figure 2:**
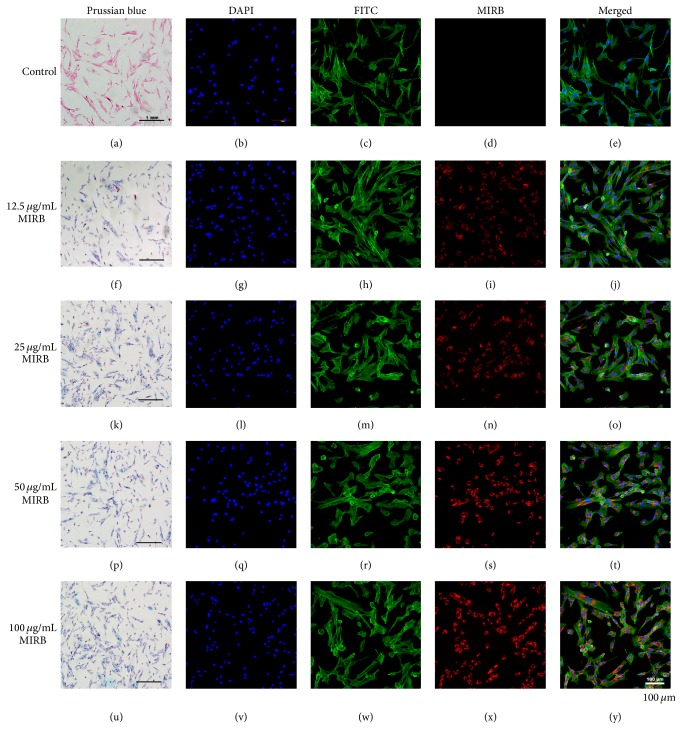
Morphological observation of hDPSCs labeled with various concentrations of MIRB. Light microscopy images: (a) Prussian blue staining of unlabeled control hDPSCs; (f, k, p, u) Prussian blue staining of 12.5 *μ*g/mL–100 *μ*g/mL MIRB-labeled hDPSCs. The scale bar indicates 1 mm. Fluorescence microscopy images: (b, g, l, q, v) the nuclei of hDPSCs are stained with DAPI (blue); (c, h, m, r, w) the cytoskeleton is stained with FITC-phalloidin (green); (d, i, n, s, x) the MIRB particles show red; (e, j, o, t, y) merged images of nuclei, cytoskeleton, and MIRB. The scale bar indicates 100 *μ*m.

After incubation with different concentrations of MIRB for 24 h, internalized MIRB was stained with Prussian blue as observed by light microscopy (Figures 2(a), 2(f), 2(k), 2(p), and 2(u)). Laser confocal microscopy was performed to detect labeling of MIRB (Figures 2(d), 2(i), 2(n), 2(s), and 2(x)) as well as nuclei (Figures 2(b), 2(g), 2(l), 2(q), and 2(v)) and cytoskeleton (Figures 2(c), 2(h), 2(m), 2(r), and 2(w)). Merged images of nuclei, cytoskeleton, and MIRB were showed in Figures 2(e), 2(j), 2(o), 2(t), and 2(y). The laser confocal microscopy revealed that intracellular MIRB particles distributed in the cytoplasm surrounding the nuclei of hDPSCs, and the fluorescence intensities in each cell gradually ascended with the increase of the concentration of MIRB for labeling. The percentage of rhodamine B-positive cells was nearly 100% for cells labeled with 12.5, 25, 50, and 100 *μ*g/mL MIRB. A similar result was observed with the percentage of positive cells that were stained with Prussian blue.

**Figure 3:**
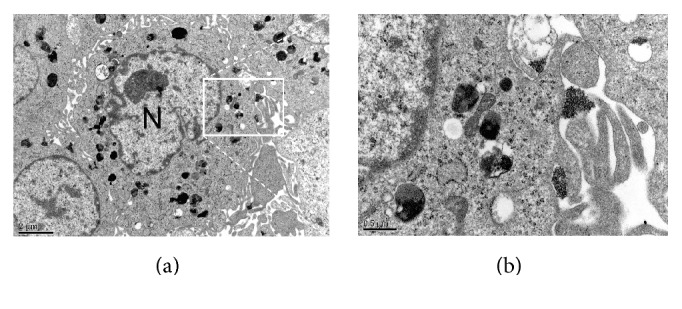
(a) and (b) TEM images of MIRB internalized in hDPSCs; (b) rRepresents several vesicles loaded with MIRB selected from the boxed area of (a). The magnification of image (b) is 40000x. The bar in image (a) is 2 *µ*m; in (b) it represents 500 nm.

To further understand where the particles are located within the cells, transmission electron microscopy (TEM) images of hDPSCs labeled with MIRB are shown in Figure 3. TEM showed that iron particles were compartmentalized within endosomes in the cell cytoplasm. The small dark spheres within the vesicles are the iron oxide core of MIRB nanoparticles.

### 3.4. Intracellular Iron Content

**Figure 4:**
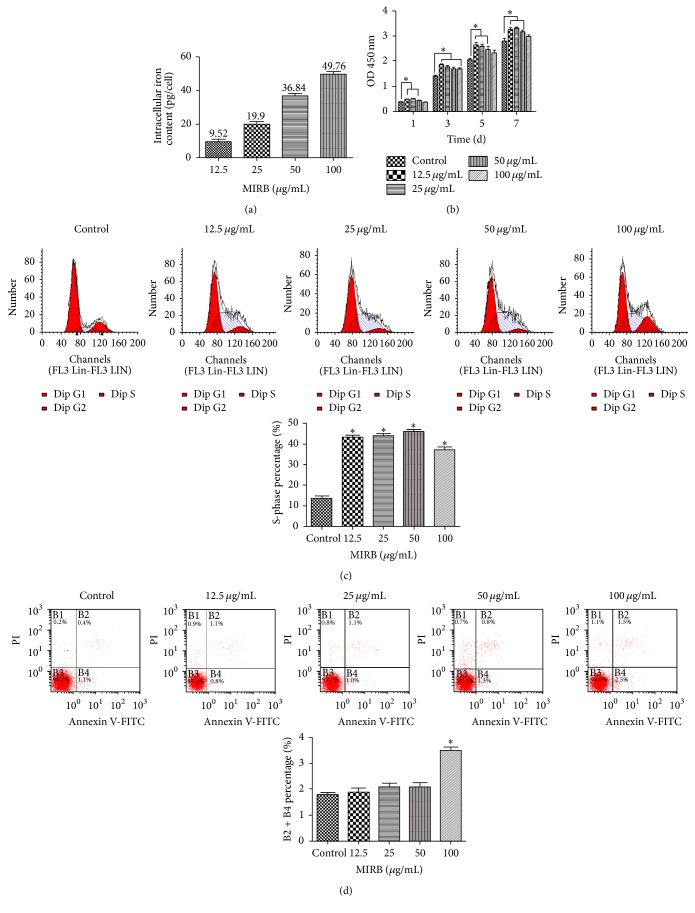
Biological effects of MIRB on hDPSCs. (a) Intracellular iron content analysis. (b) Proliferation capacity of hDPSCs measured with MTT assay. MIRB (12.5 *μ*g/mL–50 *μ*g/mL) promotes hDPSCs proliferation. ^*∗*^*P* < 0.05. (c) Promotion effect of MIRB (12.5 *μ*g/mL–100 *μ*g/mL) on cell cycle progression. ^*∗*^*P* < 0.05. (d) Effect of MIRB labeling on cell apoptosis. 100 *μ*g/mL MIRB is toxic to hDPSCs. ^*∗*^*P* < 0.05.

The intracellular iron content in 12.5 *μ*g/mL–50 *μ*g/mL groups increased in accordance with the increasing concentrations of MIRB, which were nearly 2-fold higher than the previous one. However, the intracellular iron content in 100 *μ*g/mL group was only 49.76 pg/cell, which did not reach 2-fold higher than that of 50 *μ*g/mL group (Figure 4(a)).

### 3.5. Detection of Cellular Viability of MIRB-Labeled hDPSCs

In MTT experiment, MIRB in the range of 12.5 *μ*g/mL to 50 *μ*g/mL significantly enhanced the proliferation of hDPSCs during 1 d to 7 d (*P* < 0.05), while 100 *μ*g/mL MIRB did not affect the cellular viability of hDPSCs from 5 d to 7 d (*P* > 0.05). Therefore, MIRB under 100 *μ*g/mL was safe for labeling hDPSCs and 12.5 *μ*g/mL coupled with a 24 h incubation time was a preferable choice for labeling hDPSCs (Figure 4(b)).

### 3.6. Cell Cycle and Apoptosis Assessment

After being labeled with different concentrations of MIRB (12.5 *μ*g/mL–100 *μ*g/mL) for 24 h, the S-phase percentages were much higher than that of unlabeled hDPSCs (*P* < 0.05) (Figure 4(c)), indicating that the proliferation capacity of hDPSCs was promoted after being labeled with MIRB. Meanwhile, 12.5 *μ*g/mL–50 *μ*g/mL MIRB labeling did not significantly induce cell apoptosis. However, the apoptotic rate of 100 *μ*g/mL group was higher than that of unlabeled cells, demonstrating that MIRB over 100 *μ*g/mL exhibited toxic effect on hDPSCs viability (Figure 4(d)). Therefore, 100 *μ*g/mL group was excluded for the rest of the study.

### 3.7. Differentiation Capacity

#### 3.7.1. Identification of ALP and Alizarin Red Staining

**Figure 5:**
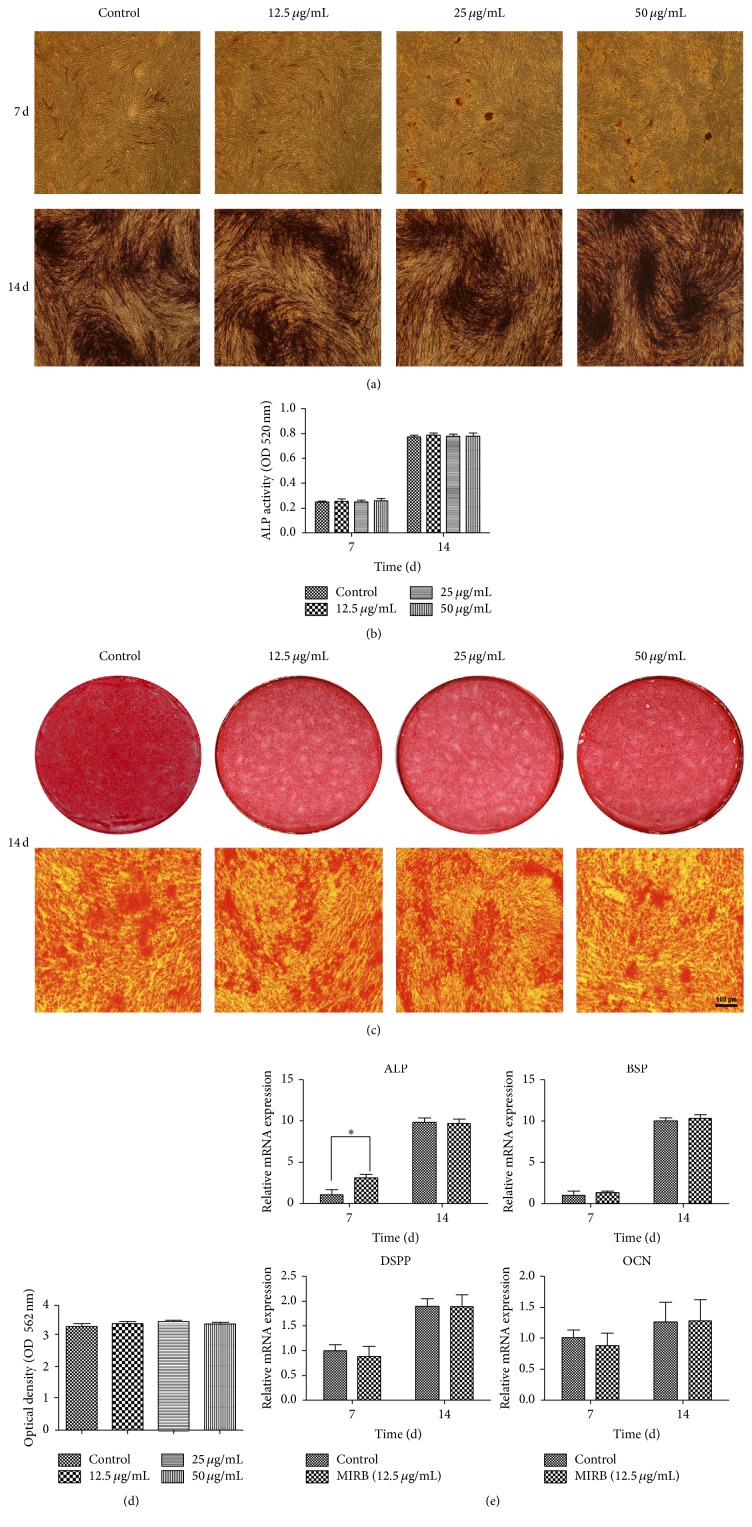
Odonto-/osteogenic differentiation analysis on MIRB-labeled and unlabeled hDPSCs. (a) Images of the ALP staining in labeled and unlabeled groups after 7 and 14 days of osteogenic induction. (b) Quantitative results of ALP staining. (c) Images of the mineral deposits of the labeled and unlabeled hDPSCs after 14 days of osteogenic induction. (d) Quantitative results of Alizarin Red staining. The scale bar indicates 100 *μ*m. (e) Expression of ALP, BSP, DSPP, and OPN by hDPSCs after 7 and 14 days of osteogenic induction. ^*∗*^*P* < 0.05.

After induction of 7 days and 14 days, the ALP activity of hDPSCs in response to different concentrations of MIRB is indicated in Figures 5(a) and 5(b). The ALP activity of all of the groups increased until day 14. However, there was no difference between MIRB-labeled groups and control group, indicating that MIRB-labeling does not affect ALP activity of hDPSCs (Figures 5(a) and 5(b)). Fourteen days after induction, the Alizarin Red staining showed that there was no difference between MIRB-labeled groups and control group (Figures 5(c) and 5(d)). Taken together, MIRB-labeling did not affect the osteogenic differentiation of hDPSCs.

#### 3.7.2. RT-PCR

The expression levels of odonto-/osteogenic genes including ALP, BSP, DSPP, and OCN were determined by RT-PCR (Figure 5(e)). At day 7, the expression level of ALP in the MIRB-labeled group was higher than that of the control group. However, there was no obvious difference on the expression of four kinds bone related genes between the MIRB-labeled group and control group at day 7 or day 14. It demonstrated that MIRB-labeling did not affect the odonto-/osteogenic differentiation of hDPSCs.

### 3.8. Magnetic Resonance Imaging of MIRB-Labeled hDPSCs In Vitro

**Figure 6:**
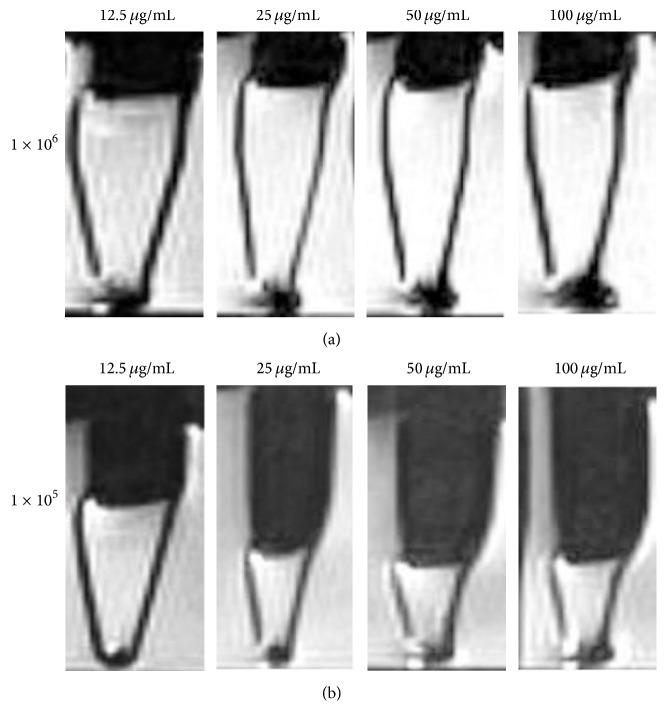
MRI of MIRB-labeled hDPSCs in vitro. (a) MRI of 1 × 10^6^ cells labeled with various concentrations of MIRB. (b) MRI of 1 × 10^5^ cells labeled with various concentrations of MIRB.

Areas containing iron-labeled cells appeared as regions of low signal intensity on Spin Echo T2-weighted MR images, creating negative contrast. The low signal regions of 1 × 10^6^ cells labeled with various concentrations of MIRB (12.5 *μ*g/mL–100 *μ*g/mL) could be visualized and the signal intensity increased with increasing concentrations of MIRB (Figure 6(a)). However, the low signal region of 1 × 10^5^ cells labeled with 12.5 *μ*g/mL was not quite obvious, but cell imaging in other groups could be easily identified (Figure 6(b)).

### 3.9. Magnetic Resonance Imaging and Histological Analysis of hDPSCs Cell Sheet In Vivo

#### 3.9.1. MRI Analysis

**Figure 7:**
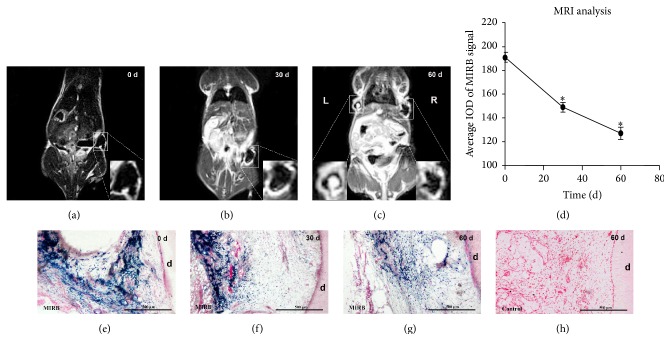
MRI and histological examination of MIRB-labeled hDPSCs cell sheets/root fragments complex in vivo. (a) In vivo MR images of nude mice immediately after transplantation. (b) In vivo MR images of nude mice 30 days after transplantation. (c) In vivo MR images of nude mice 60 days after transplantation. Left boxed area indicates the unlabeled cell sheets (high signal image) and right boxed area indicates the labeled cell sheets. (d) Quantification analysis of the signal intensity of MIRB from the boxed area of (a), (b), and (c). ^*∗*^*P* < 0.05. (e) Prussian blue staining of the MIRB-labeled group immediately after transplantation. (f) Prussian blue staining of the MIRB-labeled group 30 days after transplantation. (g) Prussian blue staining of the MIRB-labeled group 60 days after transplantation. (h) Prussian blue staining of the control group 60 days after transplantation. The scale bar of (e–h) indicates 500 *μ*m.

After MIRB-labeled and unlabeled hDPSCs cell sheets/root fragments complex were transplanted subcutaneously in the flank region, the in vivo MR images of nude mice were taken at 0 d, 1 month, and 2 months, respectively, after surgery. On 0 d, the MRI analysis showed that MIRB-labeled cell sheet showed low signal region, while surrounding soft tissue showed high signal with a clear boundary (Figure 7(a)). One month after transplantation, the low signal region became smaller (Figure 7(b)). Two months after transplantation, the low signal area became much smaller, and within which some high signal imaging could be seen (Figure 7(c) R). Compared with MIRB-labeled groups, the MR imaging of unlabeled cell sheets/root fragments complex showed high signal of the cell sheet in the middle as well as the surrounding low signal of dentin (Figure 7(c) L).

#### 3.9.2. Histological Analysis

After MRI analysis, histological examination of the implants was also performed to validate the MRI results. Prussian blue staining confirmed the presence of MIRB-labeled cells within the cell sheets surrounded by dentin (Figures 7(e), 7(f), and 7(g)) and the absence of MIRB-labeled cells in control groups (Figure 7(h)). And the amount of blue-staining cells decreased from 0 d to 60 d, which was in accordance with the MRI results.

## 4. Discussion

In recent years, with the development of tissue engineering, stem cell based therapy has become a hot spot of dental pulp regeneration [21]. The degree of success relies on two factors: first, efficient delivery and retention of dental pulp stem cells in the root canal; second, tracking the distribution, migration, and differentiation of transplanted cells in vivo. Superparamagnetic iron oxide (SPIO), as an MRI contrast agent, has been widely used in improving delivery, retention, and tracking of transplanted therapeutic cells in vivo [22].

Comparing with other MSCs, healthy and young hDPSCs can only be obtained from young permanent teeth, especially extracted impacted teeth from adults (19–29 yrs of age), so the amount of primarily cultured hDPSCs is limited. But, with several properties, such as noninvasive way to access, high proliferative potential, the capacity of self-renewal, and multilineage differentiation, hDPSCs represent a novel adult stem cell population [23], not only for dental pulp regeneration therapy but also for other stem cell based therapy, such as cardiac repair [24].

Several SPIO nanoparticles, such as Feridex® (Bayer HealthCare Pharmaceuticals Inc., Wayne, NJ, USA), have been well characterized and widely used for cell labeling and tracking by MRI [22]. Traditionally, labeling with SPIOs requires the addition of transfection reagents to the culture in order to improve uptake; however, most transfection agents cannot be applied clinically because of their cytotoxicity [25]. Furthermore, SPIO cytotoxicity varies between particle and cell type and depends on the particle coating, aggregation, and stability [26]. Insufficient coating or the release of iron into the cell can produce hydroxyl radicals (Fenton reaction) which cause DNA damage, lipid peroxidation, and protein oxidation [27–29]. In this study, we tested the ability of MIRB to label human dental pulp stem cells. We have found that incubation with various concentrations of MIRB (12.5 *μ*g/mL–100 *μ*g/mL) in the absence of transfection reagents could effectively label hDPSCs with sufficient intensity to be detected by the fluorescence of the MIRB rhodamine conjugation (Figures 2(i), 2(n), 2(s), and 2(x)). The iron oxide particle could also be detected using a Perl's iron stain (Figures 2(a), 2(f), 2(k), 2(p), and 2(u)). A recent study demonstrated that the morphology of rat bone marrow mesenchymal stem cells (rBMSCs) was not altered after being labeled with 25 *μ*g/mL MIRB [12]. Here, we also found that the morphology and structure of MIRB-labeled hDPSCs had no difference with that of unlabeled hDPSCs. Laser confocal microscopy showed that labeled and unlabeled cells were fusiform with clear cytoskeletal structures and normal pseudopodia, and it could be clearly observed that the SPIO particles were concentrated around the nuclei (Figures 2(e), 2(j), 2(o), 2(t), and 2(y)). This may indicate that MIRB-labeling will not interfere with the movements and migration of hDPSCs. Meanwhile, we also found that the fluorescence intensity ascended with the increasing of concentration of MIRB, which was also indicated by intracellular iron content analysis. However, the intracellular iron content in 100 *μ*g/mL group did not reach 2-fold higher than that of 50 *μ*g/mL group, possibly due to limited cell uptake because of Fe saturation or Fe particle clumping at high MIRB concentration [8].

Multiple types of cell have been successfully labeled with MIRB and cell vitality, proliferation, and differentiation ability were investigated [5, 6, 12, 14, 15, 30]. Shen et al. demonstrated that proliferation was unchanged between MIRB-labeled human neuroprogenitor cells [5]. Zhang et al. reported that MIRB under 100 *μ*g/mL did not affect the cellular viabilities of SD rat BMSCs [12]. On the contrary, Ren et al. demonstrated that, after labeling the BMSCs of cynomolgus monkeys with 20 *μ*g/mL MIRB, the cell proliferation decreased at passage 5 and passage 6, but there was no difference with respect to cell surface antigens and differentiation ability between MIRB-treated and untreated BMSCs [14]. Similarly, Addicott et al. reported that labeling of BMSCs of cynomolgus monkeys at MIRB concentrations of up to 30 *μ*g/mL did not affect cell vitality and proliferation, but MIRB over 30 *μ*g/mL interfere with cell vitality [15]. So the effect of MIRB on cell proliferation varies among different kind of cells. Currently, to our knowledge, there has been little study on investigating the biological effects of SPIO on hDPSCs. Only Struys et al. reported that combining 0.75 *μ*g/mL PLL with 15 *μ*g/mL SPIO (Endorem®; Guerbet, Villepinte, France) could significantly increase relative metabolic activity of hDPSCs [31]. Huang et al. demonstrated that Ferucarbotran (Schering AG, Berlin, Germany) could promote proliferation of human mesenchymal stem cell. We presumed the possible reason was that intact SPIO could diminish intracellular H_2_O_2_ through intrinsic peroxidase-like activity. Meanwhile, SPIO could accelerate cell cycle progression, which may be mediated by the free iron (Fe) released from lysosomal degradation [32]. On the contrary, Lunov et al. demonstrated that the SPIO coating could be degraded and the exposed iron oxide core would catalyze generation of reactive oxygen species (ROS), which might induce damage to cellular structures and finally cell death [33]. A recent related study about biological safety of using SPIO also demonstrated that Fe_3_O_4_-MNPs could result in significant increases in ROS production and eryptosis in the end [34]. In addition, previous study pointed out cell proliferation might be diminished as a result of cytoskeletal changes induced by the high intracellular iron oxide nanoparticles concentrations which impeded actin-mediated signaling [35]. In our present study, MIRB at 12.5 *μ*g/mL–50 *μ*g/mL could promote cell proliferation, accelerate cell cycle, but have no effect on cell apoptosis. However, 100 *μ*g/mL MIRB could accelerate cell cycle and induce apoptosis as well. Finally the proliferation in 100 *μ*g/mL group showed no difference with control group. Therefore, we presume that intracellular intact SPIO nanoparticles at low concentration can accelerate cell cycle and promote proliferation. When SPIO concentration is too high, the cell uptake has been saturated and redundant free iron may induce Fenton reaction. In addition, exposed iron oxide core may generate ROS [33], which may lead to the activation of c-Jun N-terminal kinase (JNK) pathway [36] and finally cell apoptosis [33, 37]. So, we presumed that the promotion of cell cycle and apoptosis of 100 *μ*g/mL MIRB might counteract each other and finally resulted in no effect on cell proliferation. Additionally, there was no difference regarding stem cell and hematopoietic lineage epitope patterns in unlabeled and labeled hDPSCs, indicating that stem cell characteristics were retained after MIRB labeling.

Currently, there still existed controversy about whether SPIO would affect the osteogenesis of mesenchymal stem cells. In cynomolgus monkey, untreated and MIRB-treated mesenchymal stem cells show no difference in osteogenic differentiation [14, 15]. Similar result was also found in human neural progenitor cells [5] and rat ADSCs [30]. On the contrary, Zhang et al. demonstrated that MIRB labeling inhibited the osteogenesis of rat BMSCs [12]. Clearly, the impacts of MIRB-labeling on osteogenic differentiation differ among different kinds of MSCs. During the osteogenetic experiments, we found that the cells in the MIRB-labeling group could normally induce the expressions of ALP and Ca nodules, suggesting that MIRB-labeled cells had the abilities of osteogenetic differentiation. ALP staining, alizarin red staining, and RT-PCR experiments all indicated that MIRB labeling (12.5 *μ*g/mL–50 *μ*g/mL) had no influence on osteogenic differentiation capacity.

Struys et al. pointed out that hDPSCs could be detected in 9.4 T MR scanner after being labeled with 15 *μ*g/mL SPIO, which was the optimal concentration of SPIO for labeling hDPSCs. At that moment, intracellular iron content reached 13.4 pg iron/cell, which was the minimum request for MRI detection [31]. Here, we found intracellular iron content reached 9.52 pg iron/cell at the labeling concentration of 12.5 *μ*g/mL, which was lower than the minimum request for MRI detection. In vitro MRI result showed that 1 × 10^5^ cells labeled with 12.5 *μ*g/mL could not be clearly detected in 3.0-Tesla MR scanner. However, the labeling efficiency in 12.5 *μ*g/mL group still reached 100%. Therefore, we presume that the optimal labeling concentration is not consistent. It has no relationship with cell type but has close relationship with cell condition and cell number. Additionally, the quality of MR imaging depends on the quality of MRI scanner as well.

Many studies have verified cell migration and homing by tracking transplanted cells with MRI [38–40]. Some studies demonstrated that stem cells/scaffold complex could also be detected and tracked by MRI [41, 42]. Cell sheet engineering has recently been developed as a scaffold-free strategy for cell delivery. Cell-to-cell interactions and sufficient extracellular matrix (ECM) significantly promote cell utilization efficiency and scaffold plasticity [43]. Based on so many advantages, cell sheet engineering has been widely used in tissue or organ regeneration therapy, including myocardial repair [44], corneal epithelium repair [45], and periodontium regeneration [46]. In present study, we for the first time induced MIRB-labeled hDPSCs into cell sheets and found that MIRB-labeling did not impact the generation of ECM to form cell sheet. Meanwhile, we detected the imaging of cell sheet/root fragment complex by MRI scanner and found clear low signal region of MIRB-labeled cell sheet, which decreased with the passage of time. The possible reason was that the metabolism of some cells outside the root fragment made the iron ion to be released and finally removed by phagocytosis. Another possible reason was that proliferation or differentiation of cells decreased the intracellular concentration of iron. We have laid the foundation of noninvasive observation of hDPSCs cell sheet for dental pulp regeneration in the future. However, cell differentiation condition in vivo cannot be judged only by MRI detection, which needs further histological analysis.

## 5. Conclusions

This study used multiple methods to investigate the biological changes of MIRB-labeled hDPSCs from many aspects. The cytoskeletal structures were not changed obviously after labeling. 12.5 *μ*g/mL–50 *μ*g/mL MIRB could efficiently label hDPSCs without significant effects on cell viability, phenotype and differentiation. 100 *μ*g/mL MIRB showed cytotoxicity and promote cell apoptosis. MIRB-labeled hDPSCs were detected in vitro and we found the intracellular iron content of 12.5 *μ*g/mL group was not sufficient to be detected by MRI scanner. Furthermore, we found that MIRB-labeling did not interfere with the generation of cell sheet and cell sheet/root fragment complex could be clearly detected in vivo by MRI. Our study suggests that MIRB is a useful tracer for dental pulp regeneration therapy and further in vivo histological study should be investigated.

## References

[1] Gronthos S., Mankani M., Brahim J., Robey P. G., Shi S. (2000). Postnatal human dental pulp stem cells (DPSCs) in vitro and in vivo. *Proceedings of the National Academy of Sciences of the United States of America*.

[2] Ponnaiyan D., Jegadeesan V. (2014). Comparison of phenotype and differentiation marker gene expression profiles in human dental pulp and bone marrow mesenchymal stem cells. *European Journal of Dentistry*.

[3] Isobe Y., Koyama N., Nakao K. (2016). Comparison of human mesenchymal stem cells derived from bone marrow, synovial fluid, adult dental pulp, and exfoliated deciduous tooth pulp. *International Journal of Oral and Maxillofacial Surgery*.

[4] Aurrekoetxea M., Garcia-Gallastegui P., Irastorza I. (2015). Dental pulp stem cells as a multifaceted tool for bioengineering and the regeneration of craniomaxillofacial tissues. *Frontiers in Physiology*.

[5] Shen W.-B., Plachez C., Chan A. (2013). Human neural progenitor cells retain viability, phenotype, proliferation, and lineage differentiation when labeled with a novel iron oxide nanoparticle, Molday ION Rhodamine B. *International Journal of Nanomedicine*.

[6] McFadden C., Mallett C. L., Foster P. J. (2011). Labeling of multiple cell lines using a new iron oxide agent for cell tracking by MRI. *Contrast Media & Molecular Imaging*.

[7] Guzman R., Uchida N., Bliss T. M. (2007). Long-term monitoring of transplanted human neural stem cells in developmental and pathological contexts with MRI. *Proceedings of the National Academy of Sciences of the United States of America*.

[8] Wang N., Zhao J.-Y., Guan X. (2015). Biological characteristics of adipose tissue-derived stem cells labeled with amine-surface-modified superparamagnetic iron oxide nanoparticles. *Cell Biology International*.

[9] Cheng K., Li T.-S., Malliaras K., Davis D. R., Zhang Y., Marbán E. (2010). Magnetic targeting enhances engraftment and functional benefit of iron-labeled cardiosphere-derived cells in myocardial infarction. *Circulation Research*.

[10] Song M., Kim Y.-J., Kim Y.-H., Roh J., Kim S. U., Yoon B.-W. (2010). Using a neodymium magnet to target delivery of ferumoxide-labeled human neural stem cells in a rat model of focal cerebral ischemia. *Human Gene Therapy*.

[11] Jin W., Yang X., Li Z. (2016). Non-invasive tracking of CD4^+^ T cells with a paramagnetic and fluorescent nanoparticle in brain ischemia. *Journal of Cerebral Blood Flow & Metabolism*.

[12] Zhang G., Na Z., Ren B., Zhao X., Liu W. (2015). Impacts of fluorescent superparamagnetic iron oxide (SPIO)-labeled materials on biological characteristics and osteogenesis of bone marrow mesenchymal stem cells (BMSCs). *International Journal of Clinical and Experimental Medicine*.

[13] He T., Wang Y., Xiang J., Zhang H. (2014). In vivo tracking of novel SPIO-molday ION rhodamine-B™-labeled human bone marrow-derived mesenchymal stem cells after lentivirus-mediated COX-2 silencing: a preliminary study. *Current Gene Therapy*.

[14] Ren Z., Wang J., Zou C., Guan Y., Zhang Y. A. (2011). Labeling of cynomolgus monkey bone marrow-derived mesenchymal stem cells for cell tracking by multimodality imaging. *Science China Life Sciences*.

[15] Addicott B., Willman M., Rodriguez J. (2011). Mesenchymal stem cell labeling and *in vitro* MR characterization at 1.5 T of new SPIO contrast agent: molday ION rhodamine-B™. *Contrast Media and Molecular Imaging*.

[16] Xiao W.-D., Yu A.-X., Liu D.-L. (2014). Fasudil hydrochloride could promote axonal growth through inhibiting the activity of ROCK. *International Journal of Clinical and Experimental Pathology*.

[17] Sun H.-H., Chen B., Zhu Q.-L. (2014). Investigation of dental pulp stem cells isolated from discarded human teeth extracted due to aggressive periodontitis. *Biomaterials*.

[18] Wang Z., Ding L., Zhang S., Jiang T., Yang Y., Li R. (2014). Effects of icariin on the regulation of the OPG-RANKL-RANK system are mediated through the MAPK pathways in IL-1*β*-stimulated human SW1353 chondrosarcoma cells. *International Journal of Molecular Medicine*.

[19] Chen B., Sun H.-H., Wang H.-G., Kong H., Chen F.-M., Yu Q. (2012). The effects of human platelet lysate on dental pulp stem cells derived from impacted human third molars. *Biomaterials*.

[20] Huang G. T.-J., Yamaza T., Shea L. D. (2010). Stem/Progenitor cell-mediated de novo regeneration of dental pulp with newly deposited continuous layer of dentin in an in vivo model. *Tissue Engineering A*.

[21] Kim S. G., Zheng Y., Zhou J. (2013). Dentin and dental pulp regeneration by the patient's endogenous cells. *Endodontic Topics*.

[22] Connell J. J., Patrick P. S., Yu Y., Lythgoe M. F., Kalber T. L. (2015). Advanced cell therapies: targeting, tracking and actuation of cells with magnetic particles. *Regenerative Medicine*.

[23] Gronthos S., Brahim J., Li W. (2002). Stem cell properties of human dental pulp stem cells. *Journal of Dental Research*.

[24] Gandia C., Armiñan A. N. A., García-Verdugo J. M. (2008). Human dental pulp stem cells improve left ventricular function, induce angiogenesis, and reduce infarct size in rats with acute myocardial infarction. *Stem Cells*.

[25] Arbab A. S., Yocum G. T., Wilson L. B. (2004). Comparison of transfection agents in forming complexes with ferumoxides, cell labeling efficiency, and cellular viability. *Molecular Imaging*.

[26] Gupta A. K., Gupta M. (2005). Cytotoxicity suppression and cellular uptake enhancement of surface modified magnetic nanoparticles. *Biomaterials*.

[27] Imlay J. A., Chin S. M., Linn S. (1988). Toxic DNA damage by hydrogen peroxide through the fenton reaction in vivo and in vitro. *Science*.

[28] Lewinski N., Colvin V., Drezek R. (2008). Cytotoxicity of nanopartides. *Small*.

[29] Voinov M. A., Pagán J. O. S., Morrison E., Smirnova T. I., Smirnov A. I. (2011). Surface-mediated production of hydroxyl radicals as a mechanism of iron oxide nanoparticle biotoxicity. *Journal of the American Chemical Society*.

[30] Nan H., Huang J., Li H., Li Q., Liu D. (2013). Assessment of biological characteristics of adipose tissue-derived stem cells co-labeled with Molday ION Rhodamine B™ and green fluorescent protein in vitro. *Molecular Medicine Reports*.

[31] Struys T., Ketkar-Atre A., Gervois P. (2013). Magnetic resonance imaging of human dental pulp stem cells in vitro and in vivo. *Cell Transplantation*.

[32] Huang D.-M., Hsiao J.-K., Chen Y.-C. (2009). The promotion of human mesenchymal stem cell proliferation by superparamagnetic iron oxide nanoparticles. *Biomaterials*.

[33] Lunov O., Syrovets T., Büchele B. (2010). The effect of carboxydextran-coated superparamagnetic iron oxide nanoparticles on c-Jun N-terminal kinase-mediated apoptosis in human macrophages. *Biomaterials*.

[34] Ran Q., Xiang Y., Liu Y. (2015). Eryptosis indices as a novel predictive parameter for biocompatibility of Fe_3_O_4_ magnetic nanoparticles on erythrocytes. *Scientific Reports*.

[35] Soenen S. J. H., Nuytten N., De Meyer S. F., De Smedt S. C., De Cuyper M. (2010). High intracellular iron oxide nanoparticle concentrations affect cellular cytoskeleton and focal adhesion kinase-mediated signaling. *Small*.

[36] Temkin V., Karin M. (2007). From death receptor to reactive oxygen species and c-Jun N-terminal protein kinase: the receptor-interacting protein 1 odyssey. *Immunological Reviews*.

[37] Lin A. (2003). Activation of the JNK signaling pathway: breaking the brake on apoptosis. *BioEssays*.

[38] Dekaban G. A., Hamilton A. M., Fink C. A. (2013). Tracking and evaluation of dendritic cell migration by cellular magnetic resonance imaging. *Wiley Interdisciplinary Reviews: Nanomedicine and Nanobiotechnology*.

[39] Loebinger M. R., Kyrtatos P. G., Turmaine M. (2009). Magnetic resonance imaging of mesenchymal stem cells homing to pulmonary metastases using biocompatible magnetic nanoparticles. *Cancer Research*.

[40] Long C. M., Bulte J. W. M. (2009). In vivo tracking of cellular therapeutics using magnetic resonance imaging. *Expert Opinion on Biological Therapy*.

[41] Lalande C., Miraux S., Derkaoui S. M. (2011). Magnetic resonance imaging tracking of human adipose derived stromal cells within three-dimensional scaffolds for bone tissue engineering. *European Cells and Materials*.

[42] Terrovitis J. V., Bulte J. W. M., Sarvananthan S. (2006). Magnetic resonance imaging of ferumoxide-labeled mesenchymal stem cells seeded on collagen scaffolds—relevance to tissue engineering. *Tissue Engineering*.

[43] Matsuura K., Utoh R., Nagase K., Okano T. (2014). Cell sheet approach for tissue engineering and regenerative medicine. *Journal of Controlled Release*.

[44] Shimizu T., Yamato M., Akutsu T. (2002). Electrically communicating three-dimensional cardiac tissue mimic fabricated by layered cultured cardiomyocyte sheets. *Journal of Biomedical Materials Research*.

[45] Watanabe K., Yamato M., Hayashida Y. (2007). Development of transplantable genetically modified corneal epithelial cell sheets for gene therapy. *Biomaterials*.

[46] Wang Z.-S., Feng Z.-H., Wu G.-F. (2016). The use of platelet-rich fibrin combined with periodontal ligament and jaw bone mesenchymal stem cell sheets for periodontal tissue engineering. *Scientific Reports*.

